# Delayed anterior segment complications after the treatment of retinopathy of prematurity with laser photocoagulation

**DOI:** 10.3389/fopht.2023.1270591

**Published:** 2023-11-16

**Authors:** Aparna Ajjarapu, Alina Dumitrescu

**Affiliations:** Department of Ophthalmology and Visual Sciences, University of Iowa Carver College of Medicine, Iowa City, IA, United States

**Keywords:** retinopathy of prematurity, laser photocoagulation, cataract, glaucoma, anterior segment

## Abstract

**Purpose:**

This retrospective cohort study presents a group of patients who underwent laser therapy for retinopathy of prematurity and presented with delayed anterior segment complications.

**Methods:**

The charts of infants treated with laser photocoagulation for retinopathy of prematurity at our institution between 1988 and 2020 were reviewed. The data extracted included demographics, treatment and clinical examination findings, and those on visual acuity, surgical procedures, and cycloplegic refraction. The inclusion criteria were documented anterior segment changes during the follow-up period. The exclusion criteria were any prior intraocular surgery or inflammation before signs of anterior segment complications developed. The exposure was laser photocoagulation for retinopathy of prematurity and the main outcomes were anterior segment complications, visual acuity, and cycloplegic refraction.

**Results:**

A total of 183 charts were reviewed. Sixteen eyes of nine patients (4.4%) met the inclusion criteria. The mean follow-up period after laser treatment was 15.9 years (range 10 years–26 years). The mean gestational age at birth was 24.6 weeks (range 23 weeks–27 weeks), and the mean age at first clinical documentation of anterior segment complications was 8.7 years (range 1 years–25 years). The complications included cataract (*n* = five patients/eight eyes) and glaucoma (*n* = three patients/five eyes), with the most frequent complication being band keratopathy (*n* = nine patients/15 eyes). A total of five out of 16 eyes underwent surgical procedures due to anterior segment complications. After treatment, visual acuity improved back to its baseline value in four out of five of the treated eyes, and improved, but not to its baseline value, in one out of five of the treated eyes. All the patients developed progressive high myopia over the follow-up period.

**Conclusions:**

Anterior segment complications after laser photocoagulation for retinopathy of prematurity may develop later in life. They affect patients’ visual acuity and quality of life and may require treatment.

## 1 Introduction

Retinopathy of prematurity (ROP) affects the developing retinal vasculature of premature infants and is a leading cause of childhood blindness worldwide. If left untreated, the progression of ROP can lead to retinal neovascularization, vitreous hemorrhage, retinal detachment, and permanent blindness (1).

Laser photocoagulation is recognized as one of the standard treatments for ROP (2–4). Through thermal ablation to the avascular retina, it decreases the retinal levels of vascular endothelial growth factor (VEGF), and prevents the abnormal growth of blood vessels in the retina (5). Although laser photocoagulation is highly effective and frequently used for the treatment of ROP, it has been reported to be associated with ocular complications of the anterior segment such as band keratopathy, cataracts, glaucoma, and anterior segment ischemia (ASI) (6–15). All of these complications can permanently decrease visual acuity, and their treatment might require additional procedures. ASI, a rare complication of strabismus surgery (16) and a less well-characterized complication of laser therapy, occurs when the blood flow to the anterior ciliary vessels is disrupted.

Most studies that describe anterior segment complications after laser treatment do so in the short term (i.e., < 5 years after treatment) (8–11, 15). Although prior studies have investigated the long-term functional and structural outcomes of laser therapy for ROP (17, 18), no studies have explicitly reported the delayed anterior segment complications of laser therapy for ROP. A new concept for ROP as a lifelong disease with consequences and complications extending into adulthood has been proposed (14), and this study adds to the body of evidence supporting this concept.

The present study is unique in that it aims to report delayed postoperative anterior segment complications in a cohort of patients with prematurity and threshold ROP who underwent laser therapy and were treated or referred to the Pediatric Ophthalmology Clinic.

## 2 Methods

### 2.1 Study design

A retrospective cohort study was conducted by reviewing the medical records of all premature infants with a primary diagnosis of ROP who underwent laser therapy in accordance with the *International Classification of Diseases*, Ninth Revision (ICD-9), Current Procedural Terminology (CPT) codes, and laser treatment codes at our institution from 23 February 1988 to 7 May 2020. The project protocol was reviewed and accepted by the Institutional Review Board.

### 2.2 Setting

The medical records of 183 patients were reviewed. Pertinent data including race/ethnicity, birth information (gestational age and birth weight), stage/zone of ROP at treatment, age at laser treatment, laser treatment details, age at first clinical documentation of anterior segment complications, features of anterior segment complications and procedures performed to treat it (i.e., chelation and cataract extraction), medical treatments (i.e., lubrication and IOP-lowering drops), additional procedures (i.e., retinal detachment repair and strabismus surgery), visual acuity, and cycloplegic refraction measurements during follow-up visits were recorded on a standardized form.

### 2.3 Participants and study size

This study’s inclusion criteria were infants who underwent laser therapy for threshold ROP (*n* = 183) and had documented anterior segment changes over the follow-up period (*n* = 37). The time point at which anterior segment changes were first documented in the patient’s record was considered the onset of the condition. The exclusion criteria were patients who did not have clinical signs of anterior segment complications after treatment (*n* = 146). The participants were also excluded if they had undergone any prior intraocular surgery including cataract, vitrectomy, or other procedures; had documented intraocular inflammation before the signs of anterior segment complications were recorded; or had incomplete records (*n* = 28). Out of the 183 patients who underwent laser photocoagulation for ROP, 16 eyes of nine patients met the eligibility criteria for this study.

### 2.4 Variables

The primary outcome was the presence of anterior segment complications, which was defined as an abnormal finding of the cornea, lens, or anterior chamber angle documented on the slit lamp examination and disease diagnosis related to the cornea, cataract, or glaucoma in the medical record. The procedures that were performed to treat the anterior segment complications were recorded. The secondary outcomes included the visual acuity scores and results of the cycloplegic refraction, which were recorded and performed, respectively throughout the follow-up period. The visual acuity scores were transformed to LogMAR, and the results of the cycloplegic refraction were transformed to the spherical equivalent in the final data analysis.

### 2.5 Statistical methods

The means and standard deviations were calculated when applicable. Microsoft Excel^®^ (Microsoft Corporation, Redmond, WA, USA) version 16.38 was used to conduct all statistical analyses.

## 3 Results

The 16 eyes of nine patients who met the inclusion criteria, or 4.4% of the cohort, were identified as having features consistent with anterior segment complications after the treatment of ROP with laser photocoagulation. Two eyes were excluded as the left eye [oculus sinister (OS)] of patient number 7 had intraocular surgery prior to anterior segment complications, and the OS of patient number 2 did not have any anterior segment abnormalities.

For these nine patients, the mean gestational age at birth was 24.6 weeks (range 23 weeks–27 weeks) and the mean birth weight was 663.9 g (range 462 g–980 g). The indicators of treatment being required were threshold ROP, stage III ROP, zone 2 (eight out of nine patients), and zone 1 (one out of nine patients) bilateral disease, and all nine patients underwent bilateral treatment (**Table 1**). Five patients had plus disease documented at the time of treatment, and the other four met the threshold criteria for treatment based on the number of cumulative clock hours for stage III disease. The mean gestational age at treatment was 36 weeks (range 34 weeks–37 weeks), and the mean follow-up period after laser treatment was 15.9 years (range 10 years–26 years). The average number of laser spots in the study cohort was 1,636.8 per eye (range 719 spots–1,900 spots), and the standard procedure for laser photocoagulation treatment was performed using a diode laser. Nine different surgeons (pediatric ophthalmologists or retina specialists) performed the treatment.

**Table 1:** Baseline clinical characteristics of the patients with retinopathy of prematurity (ROP) who were treated with laser therapy at [the University of Iowa Hospitals and Clinics] (1988–2020).

Patient number	Ethnicity/race	Gestational age (weeks)	Birth weight (g)	ROP stage/zone involvement (treated eye: OD, OS, or both)
1	Hispanic/Latino	24	785	Stage III and zone 2 with plus disease (both)
2	Non-Hispanic/white	23	650	Stage III and zone 2 (both)
3	Unknown/white	25	874	Stage III and zone 2 (both)
4	Non-Hispanic/white	27	980	Stage III and zone 2 with plus disease (both)
5	Non-Hispanic/white	25	462	Stage III and zone 1 (both)
6	Non-Hispanic/white	23	482	Stage III and zone 2 (both)
7	Non-Hispanic/white	25	580	Stage III and zone 2 with plus disease (both)
8	Non-Hispanic/white	25	567	Stage III and zone 2 with plus disease (both)
9	Non-Hispanic/white	24	595	Stage III and zone 2 with plus disease (both)

**Figure 1:**
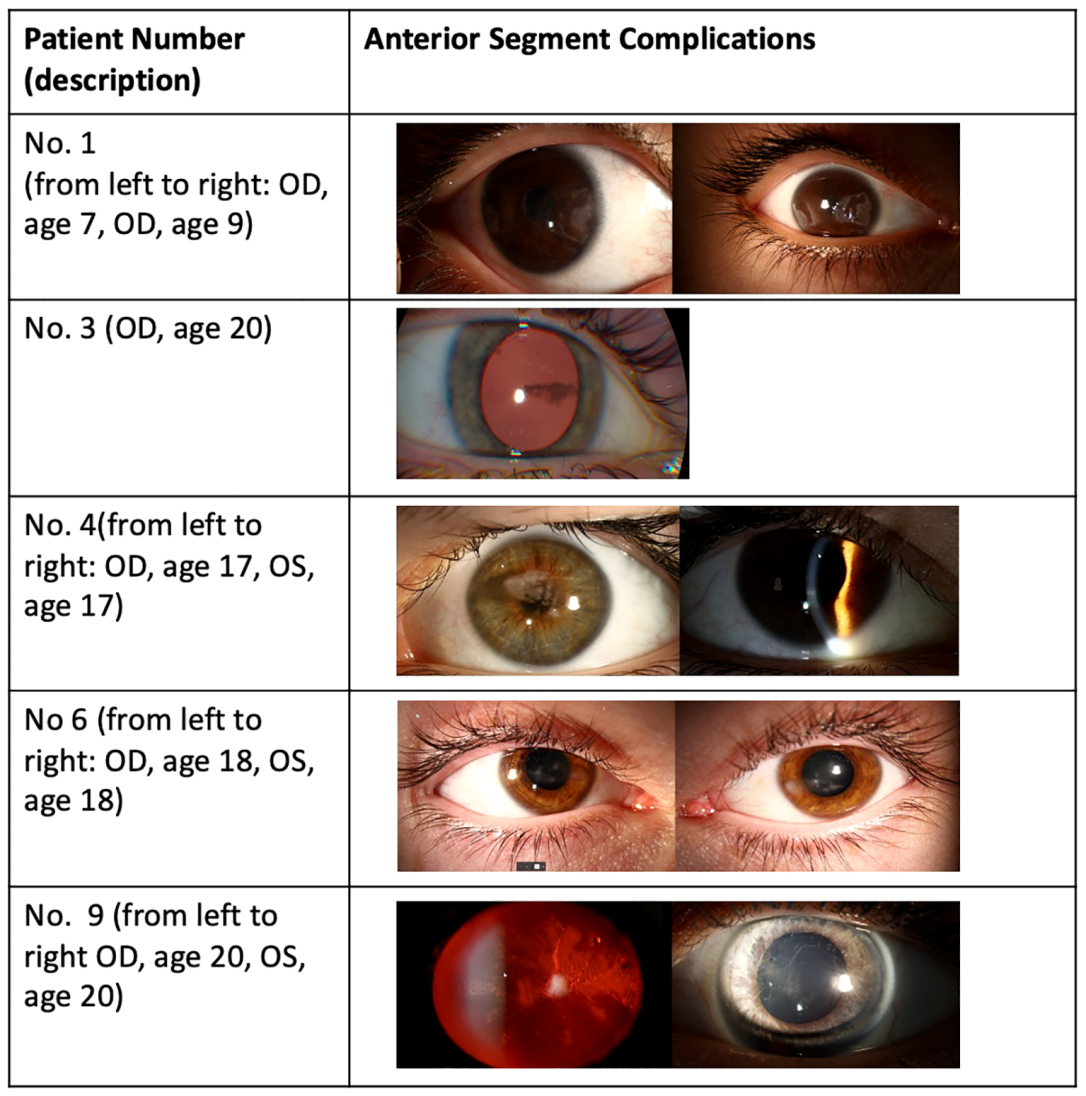
Examples of anterior segment complications in the study population.

On average, patients underwent follow-up visits once per year, and onset was considered as the first time that a clinical change was documented. The first clinical documentation of the anterior segment was delayed, with a mean age at first documentation of 8.8 years (range 1 year–25 years). The clinical symptoms of anterior segment complications included band keratopathy (*n* = 9 patients/15 eyes), cataract formation (*n* = five patients/eight eyes), glaucoma (*n* = three patients/five eyes), pupillary membrane, and posterior synechia (*n* = three patients/four eyes) (**Table 2**; **Figure 1**). Band keratopathy was the most frequent complication that came first (*n* = seven patients/10 eyes) and was progressive and worsened over time in at least two patients (**Table 2**). The time at which other complications such as cataract and glaucoma developed following band keratopathy varied from within the year of treatment to 8 years after treatment. None of the patients had intraocular inflammation documented during the follow-up period, and eight out of nine patients had documented extensive workup for systemic inflammation, which was negative.

**Table 2:** Features of anterior segment complications of patients with retinopathy of prematurity (ROP) treated with laser therapy at [the University of Iowa Hospitals and Clinics] (1988–2020).

Patient number	Age at first documentation	Features of anterior segment complications (age at documentation)	
		OD	OS
1	2 years	• Cataract (2 years of age) • Posterior synechia (3 years of age) • Band keratopathy (3 years of age) • Glaucoma (8 years of age) • Shallow anterior chamber (10 years of age)	• Few posterior synechia (3 years of age) • Band keratopathy (9 years of age) • Glaucoma (9 years of age)
2	25 years	Band keratopathy (25 years of age nasally)*	• Excluded
3	10 years	• Band keratopathy (10 years of age) • Glaucoma (16 years of age) • Shallow anterior chamber (18 years of age)	• Glaucoma (16 years of age) • Cataract (16 years of age) • Shallow anterior chamber (18 years of age)
4	12 years	• Band keratopathy (12 years of age)	• Band keratopathy (12 years of age)
5	5 years	• Glaucoma (5 years of age) • Band keratopathy (5 years of age) • Cataract (8 years of age)	• Cataract (7 years of age) • Band keratopathy (10 years of age)
6	4 years	• Band keratopathy (mild at 4 years of age) • Cataract (12 years of age)	• Band keratopathy (5 years of age) • Cataract (12 years of age)
7	1 year	• Band keratopathy (1 year of age) • Pupillary membrane (1 year of age)	• Excluded
8	13 years	• Band keratopathy (13 years of age)	• Band keratopathy (13 years of age)
9	7 years	Cataract (9 years of age) • Band keratopathy (10 years of age)	• Band keratopathy (7 years of age) • Pupillary membrane (7 years of age) • Cataract (10 years of age)

All patients experienced some degree of visual impairment after the onset of anterior segment changes. On average, patients lost 0.22 logMar units from their baseline visual acuity, representing approximately two lines on the Snellen chart. The non-surgical treatments for anterior segment complications included lubrication drops and ointments, topical steroid medication for ocular pain, and intraocular pressure-lowering mediations.

Several patients underwent additional intraocular procedures. Two patients (patient 3 at 18 years of age and patient 5 at 5 and 22 years of age) underwent glaucoma (IOP-lowering) procedures in both eyes. One patient (patient 5 at 5 and 22 years of age) underwent cataract extraction surgery bilaterally. It was observed that five patients (patient 1 at 3 years of age, patient 2 at 2 years of age, patient 4 at 2 years of age, patient 6 at 5 years of age, and patient 9 at 1 year of age) underwent strabismus surgery during the follow-up period in both eyes, an average of 2.6 years after laser surgery, with three patients (i.e., patients 2, 4, and 9) having strabismus surgery before the first signs of anterior segment complications were noted, and two patients (i.e., patients 1 and 6) having it afterwards. The majority of patients were first noted to have anterior segment complications incidentally on routine follow-ups, with only three out of nine patients presenting with symptoms such as blurred vision. All patients were followed up after the laser treatment until the complete resolution of their ROP. No patient required additional treatment (laser or vitrectomy) in the neonatal period. No additional eye abnormalities including anterior segment or retinal, other than resolved ROP were documented. All patients developed progressive high myopia, and two experienced retinal detachment (patient 1 at 4 years of age and patient 8 at 15 years of age). Their anterior segment changes were documented before developing retinal detachments (at age 2 years for patient 1, and at age 13 years for patient 8).

Five out of 16 eyes underwent additional corneal surgical procedures due to anterior segment complications (**Table 3**). Visual acuity improved after treatment in four out of five of the treated eyes and did not improve back to baseline in one out of five of the treated eyes. One eye, which underwent multiple procedures, eventually lost vision to light perception [patient 5 oculus dexter (OD)]. Eleven eyes did not receive corneal procedures despite exhibiting changes such as band keratopathy. The treating physicians made the decision based on the nature of the corneal disease (fibrotic haze), the risks being greater than the potential benefits, the patient not experiencing a significant decrease in their vision, and the potential for decreased vision due to macular dragging or amblyopia.

**Table 3:** Procedures to address the anterior segment changes in patients with retinopathy of prematurity (ROP) treated with laser therapy at the [the University of Iowa Hospitals and Clinics] (1988–2020).

Patient no.	Procedure to address anterior segment changes, OD, OD, or both (age at the time of procedure)
1	• Superficial keratectomy with EDTA chelation, OD (9 years of age)
2	• None
3	• None
4	• Superficial keratectomy with EDTA chelation, OD (16 years of age)
	• Phototherapeutic keratectomy, OS (18 years of age)
5	• Cataract extraction with posterior lens implantation, OS (7 years of age)
	• Cataract extraction with posterior lens implantation, OD (8 years of age)
	• EDTA chelation and posterior capsule membranectomy OD (8 years of age)
	• Superficial keratectomy OD (10 years of age)
	• Gunderson conjunctival flap, OD (20 years of age)
6	• None
7	• None
8	• None
9	• None

All patients developed progressive myopia during follow-up, with a mean cycloplegic spherical equivalent refractive error at the last follow-up visit of −14 D (range −6.5 D to −24 D). All refractive errors were corrected with glasses or contact lenses. Due to band keratopathy and corneal irregularities, some patients became intolerant to their contact lenses, and this affected both their quality of life and best corrected visual acuity.

## 4 Discussion

In this retrospective study, 16 eyes of nine patients among a cohort of 183 patients who underwent laser photocoagulation for ROP experienced delayed anterior segment complications at an average age of 8.7 years at first clinical documentation of symptoms. The anterior segment changes were visually significant, and their treatment required additional procedures in some patients. The most frequent anterior segment complication was band keratopathy. In addition, all patients experienced progressive myopia (a known consequence of laser therapy for ROP) (19, 20).

Although anterior segment complications after treatment for ROP have been previously reported in the short term (9, 15, 21, 22), complications in the long term have not been reported. The postulated mechanisms underlying the association between laser therapy and anterior segment complications include laser injury destroying major nutrient branches—such as the long posterior ciliary arteries—or scleral depression during the exam, which impairs the blood flow to the anterior segment, causing intermittent ASI (15, 23). ASI has been reported to result in complications, such as band keratopathy, cataract, and glaucoma, and previous studies have also reported that it leads to retinal detachment (12, 15, 16, 21, 22), which is consistent with the findings of our study. It is also important to consider other factors that may cause anterior segment complications other than laser therapy, such as inflammation associated with ROP or laser treatment at the time of the procedure. Localized hypoperfusion of the anterior segment due to decreased blood flow (before collaterals develop) is the leading cause of anterior segment ischemia. In older adults, ASI is associated with decreased perfusion due to common and internal carotid artery stenosis, atherosclerosis, inflammatory vascular diseases, trauma, or periocular and intraocular surgical procedures. In premature babies, ASI is described most commonly after laser treatment for ROP due to presumed laser injury to the long posterior ciliary arteries. Additional contributing factors have been proposed, including scleral depression and confluent tissue ablation (9), prenatal exposure to cocaine, and vascular abnormalities. The symptoms of ASI can include corneal edema, posterior synechiae, cataract formation, ocular hypo perfusion, or glaucoma. Other factors that have been noted to cause anterior segment complications, such as cataracts, include thermal injury to the lens material and capsule, which causes lenticular changes (6, 24). The proposed mechanisms underlying the association between glaucoma development after the laser treatment of ROP include ciliary body edema or choroidal congestion (from thermal injury to the choroidal vasculature, which results in vascular occlusion), causing anterior displacement of the lens–iris diaphragm, resulting in the narrowing of the anterior chamber angle (25, 26). Ischemia causing posterior synechiae and/or cataract formation can narrow the anterior chamber angle or impair the aqueous humor circulation. The eyes of prematurely born babies tend to have an increased lens-to-axial length ratio, with relatively anteriorly displaced lenses and steeper corneas (27). These tend to be additional risk factors for the development of glaucoma. Chronic intraocular inflammation and intraocular surgeries have been associated with the development of anterior segment pathology; however, we avoided the potential for confounding by these factors by excluding the patients who had inflammation or who had undergone intraocular surgery.

The prevalence of delayed anterior segment complications was 4.4% in our cohort, consistent with that in the literature, with studies reporting prevalence rates of anterior segment complications in the short term ranging from 2.3% to 10.8% (15, 22). In addition, the features of delayed anterior segment complications that were reported in our study are consistent with the studies reporting anterior segment complications in the short term, which included band keratopathy, cataracts, elevated intraocular pressure, narrower anterior chamber angle (13), and pupillary membrane (9, 15, 21, 22).

All these complications have the potential for additional vision loss and affect patients’ quality of life. The patients in this study had decreased vision due to the above complications and some required additional procedures for the treatment of these. Some of them became intolerant to their contact lenses, and all required some form of medical treatment.

One limitation of our study is that it was retrospective, which prevented us from accounting for all potential confounding factors. However, we minimized the effect of major confounding variables by excluding patients with inflammation or prior intraocular surgery before their first clinical documentation of anterior segment complications. It should also be noted that ASI has been reported as a rare but serious complication of strabismus surgery. It is more likely to occur in simultaneous or staged procedures involving multiple-muscle surgery on the same eye (16, 28) and in patients with cardiovascular risk factors such as vascular disease, diabetes mellitus, hyperviscosity, hemoglobinopathies, and encircling scleral buckling. Ischemia is caused by perturbation of the blood flow to the anterior segment when the muscles from the sclera are disinserted because the anterior ciliary vessels contribute to the anterior segment’s blood supply. Possible confounding from strabismus surgery should be considered, as three patients in our study cohort underwent this procedure before the anterior segment changes were documented. However, these patients had had uncomplicated strabismus surgery on one muscle in a given eye, meaning that ASI was highly unlikely to be secondary to strabismus surgery. All patients had multiple eye examinations after their laser treatment. We considered the onset of the anterior segment abnormality as the time that it was first documented in the patient’s medical records. It should be noted that an examination bias may have been involved and subtle changes could have been missed during the examination in young children, who exhibited a low level of cooperation during the slit lamp examination. Finally, the small sample size of our cohort may limit the generalizability of the results, and as such large, multicenter prospective studies may be needed in the future.

In conclusion, in this retrospective study, we describe the first-time late-onset anterior segment complications after the treatment of ROP with laser photocoagulation. Delayed anterior segment complications are not common, but they affect vision and may require additional procedures for treatment. They result in significant and sometimes permanent vision loss and affect patients’ quality of life. As such, long-term follow-up of these complications may be needed for their timely management and treatment. Treating physicians should include the potential for delayed anterior segment complications in the informed consent forms for laser photocoagulation for ROP.

## Data availability statement

The raw data supporting the conclusions of this article will be made available by the authors, without undue reservation.

## Ethics statement

This study involving humans was approved by University of Iowa Institutional Review Board (IRB #202004728).

## Author contributions

AA: Writing – original draft. AD: Writing – review & editing.
